# Liver microstructure and biochemical biomarkers in *Mormyrus kannume* from the River Nile

**DOI:** 10.1038/s41598-026-51996-9

**Published:** 2026-05-13

**Authors:** Aya Ali, Hanem Saad Abdel-Tawab, Ekbal Tadros Wassif, Alaa El-Din H. Sayed

**Affiliations:** 1 Department of Zoology, Faculty of Science, Assiut University, Assiut, 71516 Egypt; 2 Department of Molecular Biology, Molecular Biology Research and Studies Institute, Assiut University, Assiut, 71516 Egypt

**Keywords:** *Mormyrus kannume*, Biochemical parameters, Liver histology, Histochemistry, Melanomacrophage centers, Nephrology, Zoology

## Abstract

*Mormyrus kannume* were collected from the Nile River in Assiut Governorate. Then, the fish were kept at approximately 26–28 °C with a 12 h/12 h light-dark cycle in a tank (100 L) for two weeks to acclimatize to laboratory conditions. Despite the economic importance of *Mormyrus kannume* in African freshwater fisheries, especially in the Nile River, no prior studies have documented its baseline morphohistology of key organs, such as the liver, or biochemical parameters or health biomarkers. This study fills this gap by characterizing normal hepatic architecture, liver enzyme activities, and histochemical components in wild Nile River specimens, providing a broad view of the fish’s health status and helping researchers in their future studies. Hepatocytes exhibited cord-like arrangements with vesicular nuclei, intermingled with sinusoids of different shapes. Melanomacrophage centers varied in shape, location, and pigmentation. These findings establish reference physiological biomarkers for health assessment and future research on this species.

## Introduction

The Nile River is typically considered the longest river in the world. It stretches over 6800 km throughout North-Eastern Africa^69^. Elephant-snout fish, *Mormyrus kannume*^37^, is one of the most fascinating and interesting fish in the Nile. Anomah Umm Baouez is the popular name for *M. kannume* in Egypt^73^. It belongs to the family Mormyridae and the order Osteoglossiformes, and is widely distributed in Africa. Fish in this taxonomic group are often carnivorous and primarily consume aquatic insects, small crustaceans, and other invertebrates as their primary food sources^7^. *M. kannume* is nocturnal, staying on the bottom during the day, but it becomes extremely active after dark, looking for food and being connected to rocks^59^. In most running streams, *M. kannume* is a valuable part of the artisanal fishery and has great economic and commercial importance, and is marketable when caught^6^. It exhibits an elongated, laterally compressed body covered with tiny cycloid scales^51^.

Biochemical parameters are a useful tool for evaluating the health of fish, as they can provide valuable insights into their physiology and various feeding patterns, and detect signs of toxicity^5,35,82^. Additionally, liver enzyme levels, such as aspartate aminotransferase (AST), alanine aminotransferase (ALT), and alkaline phosphatase (ALP), may reflect the health of the fish^39^.

The liver is the largest internal and glandular organ in the body. Fish liver is a key organ that performs and controls several functions, including detoxification, deposition and metabolism of fat and carbohydrates, and protein synthesis, such as vitellogenin^23,59^. So, it is the most intriguing organ for assessing fish population health, which reflects the overall health of an aquatic ecosystem^18,96,99^. In the majority of teleost fish, the liver is reddish-brown and situated ventrally in the cranial region of the body cavity^23^.

Additionally, fish liver contains distinctive clusters of cells known as melanomacrophage centers (MMCs), which carry pigments such as hemosiderin, melanin, and lipofuscin^29,42^. MMCs change in size or number under stressful conditions and have been proposed as indicators of water quality^8^. Roles of MMCs in fish include immune defense by phagocytosis, destruction, and detoxification, and their use as bioindicators of environmental pollution^8,17^.

Previous studies on Nile River fish, particularly *Mormyrus kannume*, primarily examined reproduction, feeding habits, environmental impacts, and morphometric parameters such as length-weight relationships, age, growth rates, and condition factors (Khallaf and Authman, 2012; Kramer, 2013;^73^; Farrag, 2022). However, three critical research gaps remain unaddressed for this economically important species: (1) No baseline biochemical profiles exist for *M. kannume* liver function (including glucose, total protein, cholesterol) despite heavy metal pollution in the Nile; (2) no reference liver morphohistology is available for pathology diagnosis; (3) no histochemical studies have been established for health monitoring; and (4) MMCs, key immune response indicators, remain uncharacterized in this species despite their importance for pollution stress assessment. These gaps severely limit fishery management, aquaculture development, and environmental impact assessment in the Nile River, Assiut Governorate. The Nile River faces increasing anthropogenic pollution, yet without species-specific healthy liver baselines, researchers cannot distinguish normal variation from pathological changes. This study fills these critical gaps by providing comprehensive biochemical parameters, normal liver histology references, histochemical studies, and the first morphological characterization of MMC using light microscopy.

## Materials and methods

### Fish samples

*Mormyrus kannume* fish (24 specimens) were carefully collected from the Nile River, Assiut Governorate, Egypt, with the length (31 ± 0.77 cm, range: 29–33), and the weight (253.83 ± 4.24 gm, range: 240–270), placed in plastic boxes partially filled with their natural water and transferred into laboratory of Fish Biology and Pollution, Department of Zoology and Entomology, Faculty of Science, Assiut University. Then, 24 specimens were kept at 26–28 °C, pH 7.3, and 34.47 mg/L DO, with a 12 h/12 h light-dark cycle, in three tanks (100 L each) for two weeks to acclimatize to laboratory conditions prior to experiments. Water temperature, pH, and dissolved oxygen concentration (DO) were measured daily. Fish were fed with commercial fish food at 5% body weight twice a day. Approximately 20% of the water in each tank was exchanged daily throughout the acclimation period.

### Blood collection and biochemical parameters

A total of 24 fish were caught from tanks and anesthetized with a 200 ppm solution of clove powder^45^. Blood samples were taken from the caudal veins of six fish, left to clot in clean, dry centrifuge tubes at room temperature, and then centrifuged at 4000 rpm and 4 °C for 15 min^22^, and the serum was separated for biochemical assessments. A spectrophotometer (Bioanalytic Diagnostic Industry, Co.) was used to analyze serum biochemical parameters. Glucose was determined by the GOD-PAP method without deproteinization, using ready-made kits from SPINREACT, as described by Young^101^. AST, ALT, and ALP were determined by a colorimetric test using ready-to-use kits provided by Spectrum according to Young^100^. Using the direct Biuret method, serum total protein was measured^63^, and the cholesterol oxidase-peroxidase (CHOD-POD) method was used to determine the total cholesterol^12^.

### Histological and histochemical studies

All fish (24 fish) were freshly dissected, and small samples of liver were fixed in formal acetic alcohol (comprising 10 ml of 40% formaldehyde, 85 ml of absolute ethanol, and 5 ml of glacial acetic acid) for 24 h. After fixation, samples were extensively rinsed in 70% ethanol for three consecutive 24-hour periods to remove residual fixative before further processing. The samples were dehydrated through a graded ethanol series (80%, 90%, and absolute ethanol; two changes), cleared in methyl benzoate (three changes over three days), subsequently immersed in melted paraffin wax for a total of 3 h (three changes, 1 h/change), and then embedded in paraffin wax^1,95^. Sections were cut at a thickness of 4–5 μm using a microtome (Leica RM2125 RTS). Small parts of fish liver were taken for Semithin sections as soon as possible, and fixed in 2% glutaraldehyde, washed in cacodylate buffer, and then postfixed in 1% osmium tetroxide. The specimens were dehydrated using alcohol upgrades before being embedded in an epon and araldite mixture. Sections were cut at 1 μm thickness and stained with toluidine blue (pH 4.5)^57^.

Harris’ hematoxylin and eosin (H&E) stain was used to describe general histological structures^16^. Histochemical stains include the picrosirius red method for the investigation of tissue collagen^26^, orcein stain for elastic fibers, Feulgen reaction for deoxyribonucleic acids (DNA), and periodic acid Schiff’s reagent (PAS) for polysaccharides and glycogen. To verify the presence of glycogen, a control test was conducted by incubating human saliva at 37 °C for 30 min. Perls technique was used to identify melanomacrophage pigments, namely, hemosiderin, lipofuscin, and melanin^30,60^. These sections were examined and photographed using an Olympus BX51 light microscope (Olympus Corp., Japan) in conjunction with an ARTCAM-150 PIII digital camera (X40, X100)^32^.

### Statistical analysis

The basic statistics, means, standard errors, and ranges (minimum and maximum) of the measured parameters were recorded by the IBM-SPSS package version 27^54^.

## Results

### Biochemical parameters

The basic data of biochemical parameters of fish are listed in Table 1.

**Table 1:** Serum glucose, total protein, and total cholesterol levels, and liver enzyme activities.

Parameters	Mean ± SE	Minimum–Maximum
Glucose (Mg/dl)	65.50 ± 1.04	60.48**–**67.68
Total protein (Mg/dl)	3.55 ± 0.06	3.33**–**3.69
Total cholesterol (Mg/dl)	188.4 ± 0.5	186.30**–**189.90
AST (µ/l)	31.08 ± 0.66	28.89**–**32.58
ALT (µ/l)	14.97 ± 0.37	13.95**–**15.93
ALP (µ/l)	42.85 ± 1.14	39.33**–**46.26

### Histological studies

**Fig. 1:**
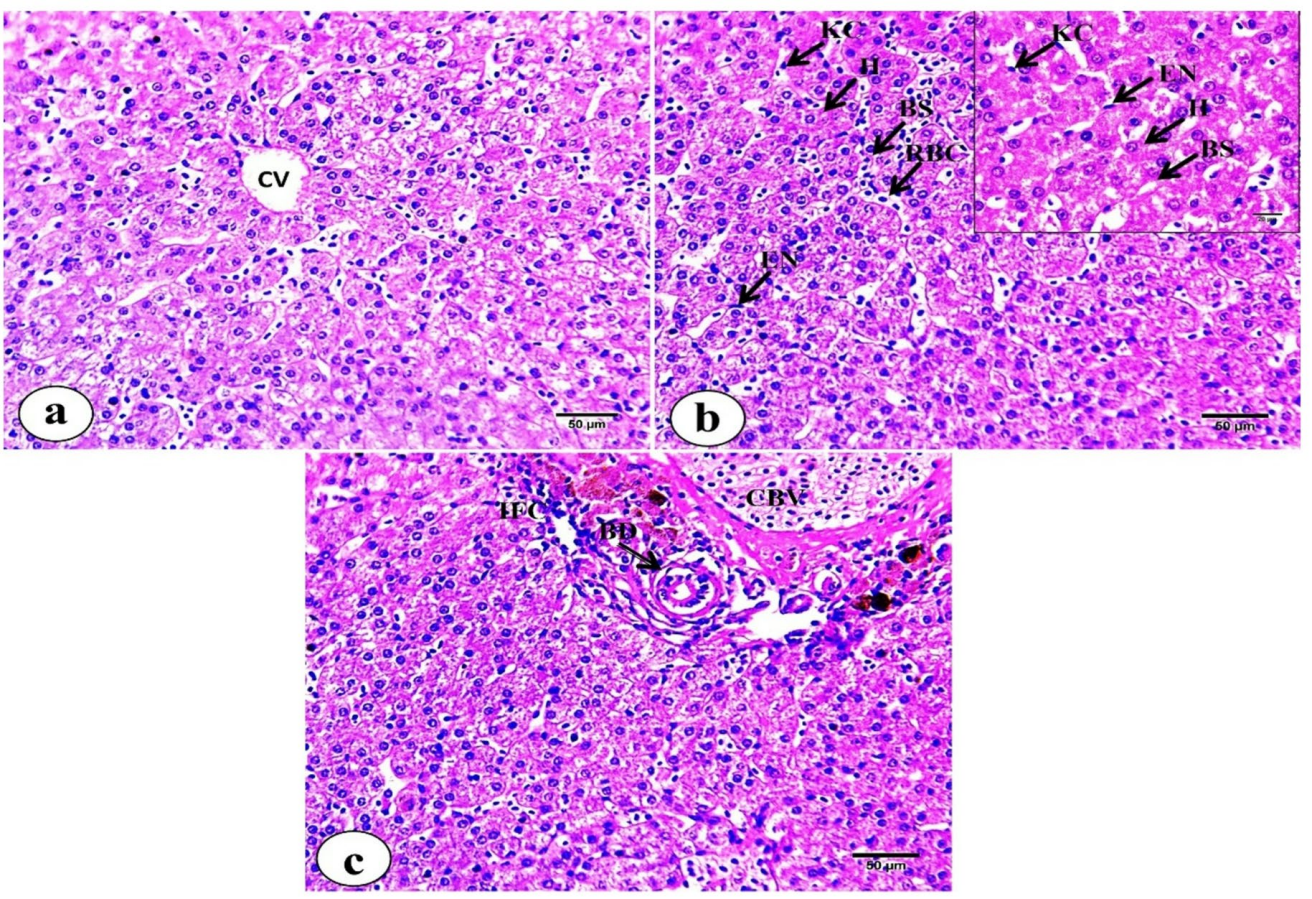
Photomicrographs of liver tissue sections of *M. kannume* stained with H&E showing: (**a**) hepatocytes arranged in cord-like strands. Central vein (CV), (**b**) hepatocytes (H), blood sinusoids (BS), endothelial cell (EN), Kupffer cell (KC), (**c**) bile duct (BD), congested blood vessel (CBV), inflammatory cells (IFC), and melanomacrophage center (MMC). (X40- Insert X100).

Hepatic tissues of *M. kannume* revealed that polyhedral hepatocytes are arranged in anastomosing laminae or cord-like strands surrounding the central vein and portal areas **(**Fig. 1a**)**. Hepatocytes exhibit homogeneous, granular, and acidophilic cytoplasm containing centrally positioned nuclei. Irregular blood sinusoids are lined by endothelial cells with flattened nuclei and Kupffer cells whose nuclei protrude into the sinusoidal lumen. The sinusoids contain mainly nucleated red blood corpuscles **(**Fig. 1b**)**. Portal areas contain bile ducts lined by cuboidal epithelial cells exhibiting acidophilic cytoplasm and large, rounded nuclei. Additionally, inflammatory cells are observed. A congested blood vessel is present, lined by endothelium and encircled by a thick sheath of acidophilic connective tissue. Few melanomacrophage cells (MMs) are also observed **(**Fig. 6c**)**.

**Fig. 2:**
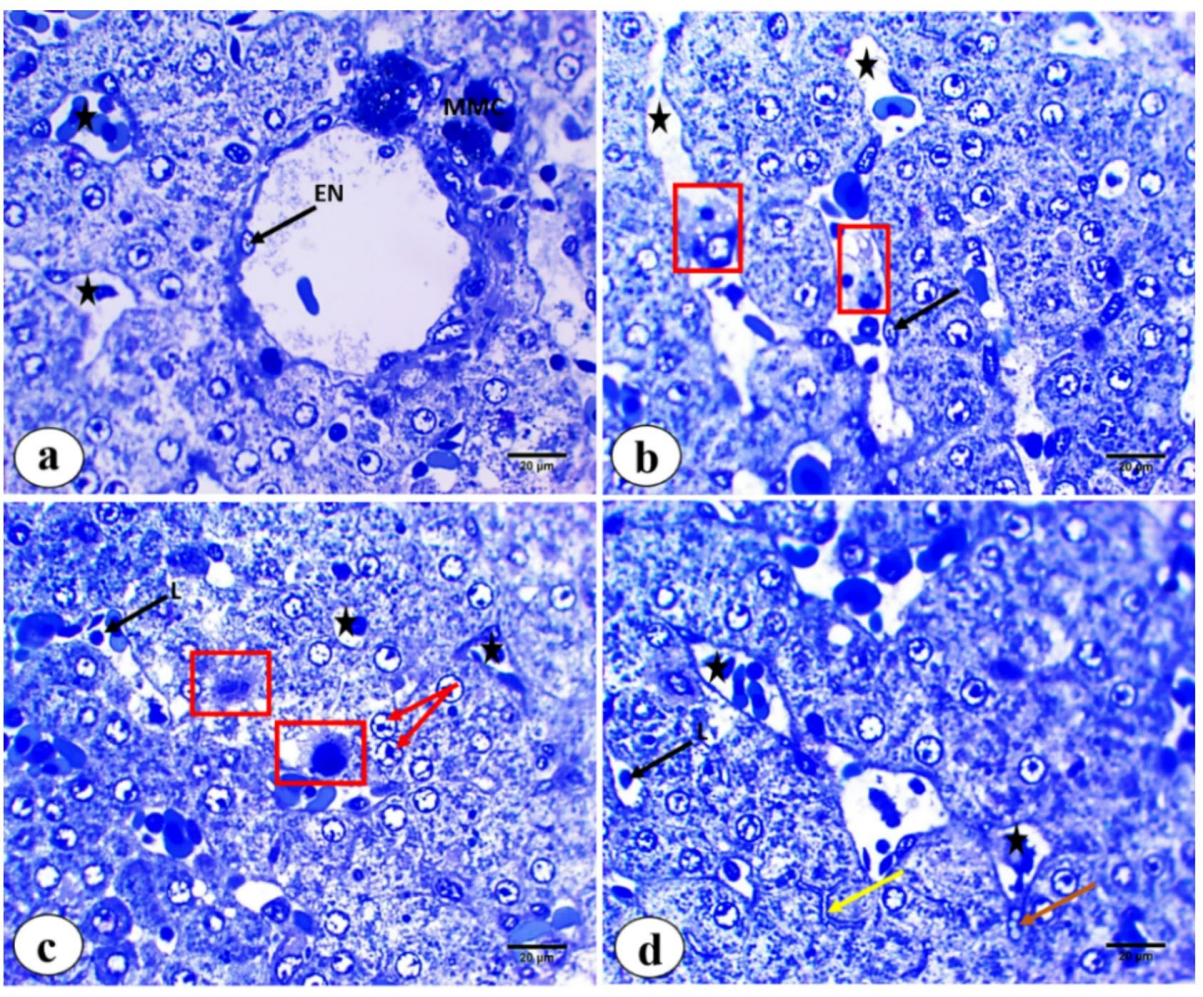
Semithin sections of *M. kannume* liver stained by toluidine blue (pH4.5) showing: **(a-d)** endothelium (black arrow), different shapes of sinusoids (stars), Kupffer cells; branched cytoplasm with long process **(b)**, large macrophage cells with irregular short process including large dark nuclei **(c)** (red square), binucleated (red arrow), biliary canaliculi (yellow arrow), Ito cell (orang arrow), melanomacrophage center (MMC), and lymphocytes (L) (X100).

Figure 2a and d show semithin sections stained with toluidine blue, where hepatocytes have cytoplasm with numerous fine blue granules; their nuclei are rounded, vesicular, with prominent nucleoli. Some cells were binucleated. MMCs were located beside central veins. Blood sinusoids were cut in various directions, taking rounded, elongated, and irregular shapes; they showed flat nuclei, most of which were related to endothelial cells. Moreover, lymphocytes were found in the blood sinusoids, with darkly stained, rounded nuclei. Kuffer phagocytic cells were observed with different shapes; some cells displayed branched cytoplasm with long processes, while others were large macrophages with large dark nuclei and irregular pseudopodia. A few pear-shaped cells containing fat droplets, which are called Ito cells, could be observed. Biliary canaliculi were also observed between hepatocytes.

**Fig. 3:**
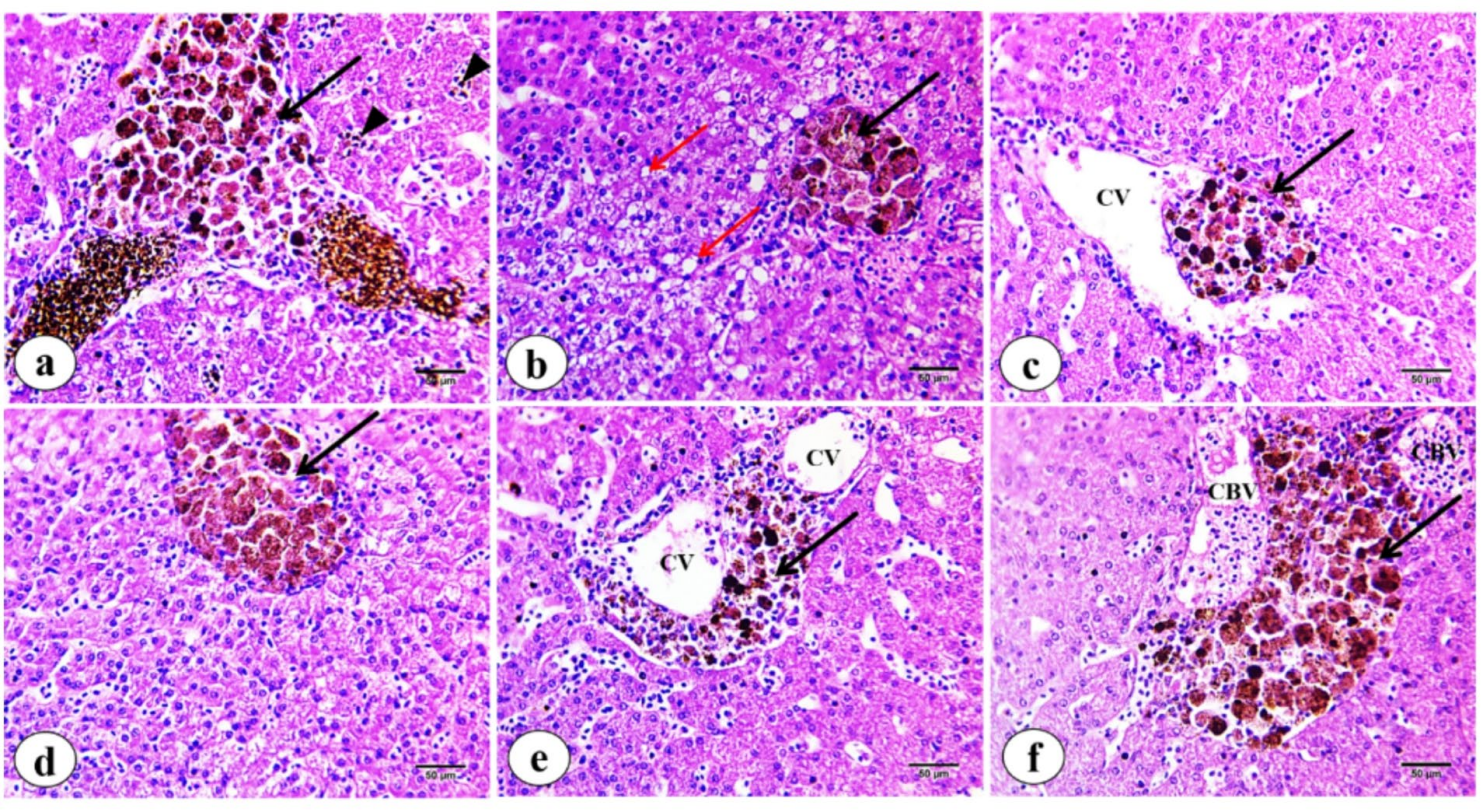
Photomicrographs of liver tissue sections of *M. kannume* stained with H&E showing melanomacrophages (black arrows) displaying different sizes, shapes, and distributions of MMC in the liver. **a)** Y-shaped MMC with aggregations of large and small pigments. Scattered small pigments distributed within blood sinasoides (head arrows). **(b)** Rounded-shaped MMC. Small and large fat droplets (red arrows). **(c)** Rounded MMC is protruding into the lumen of the central vein (CV). **(d)** Pear-shaped MMC. **(e)** Curved MMC present between two central veins. **(f)** Curved MMC positioned adjacent to congested blood vessels. Congested blood vein (CBV) (X40).

MMCs in hepatic tissues exhibited diverse morphologies and distributions, characterized by variably pigmented granules. Histological examination with hematoxylin and eosin (H&E) staining revealed that MMs constitute the predominant cellular component of MMCs, with pigmentation ranging from yellow and brown to black **(**Fig. 3a and f**)**. Notably, delta-shaped (Y-shaped) MMCs are composed of aggregations of large, pigmented cells, with smaller heterogeneous cells localized at the termini of the “Y,” predominantly containing dark pigments. Scattered pigmented cells are seen within the blood sinusoids **(a)**. Small, rounded MMCs are identified **(b)**. Furthermore, small, rounded MMCs are identified protruding into the lumen of the central vein **(c)**. Pear-shaped MMCs are noted to be incorporated within hepatocytes **(d)**, while irregular or curved (C-shaped) MMCs are situated between two central veins **(e)** or positioned adjacent to congested blood vessels, which are engorged with erythrocytes **(f)**. In addition, hepatocytes exhibit hydropic and fatty degeneration.

### Histochemical studies

**Fig. 4:**
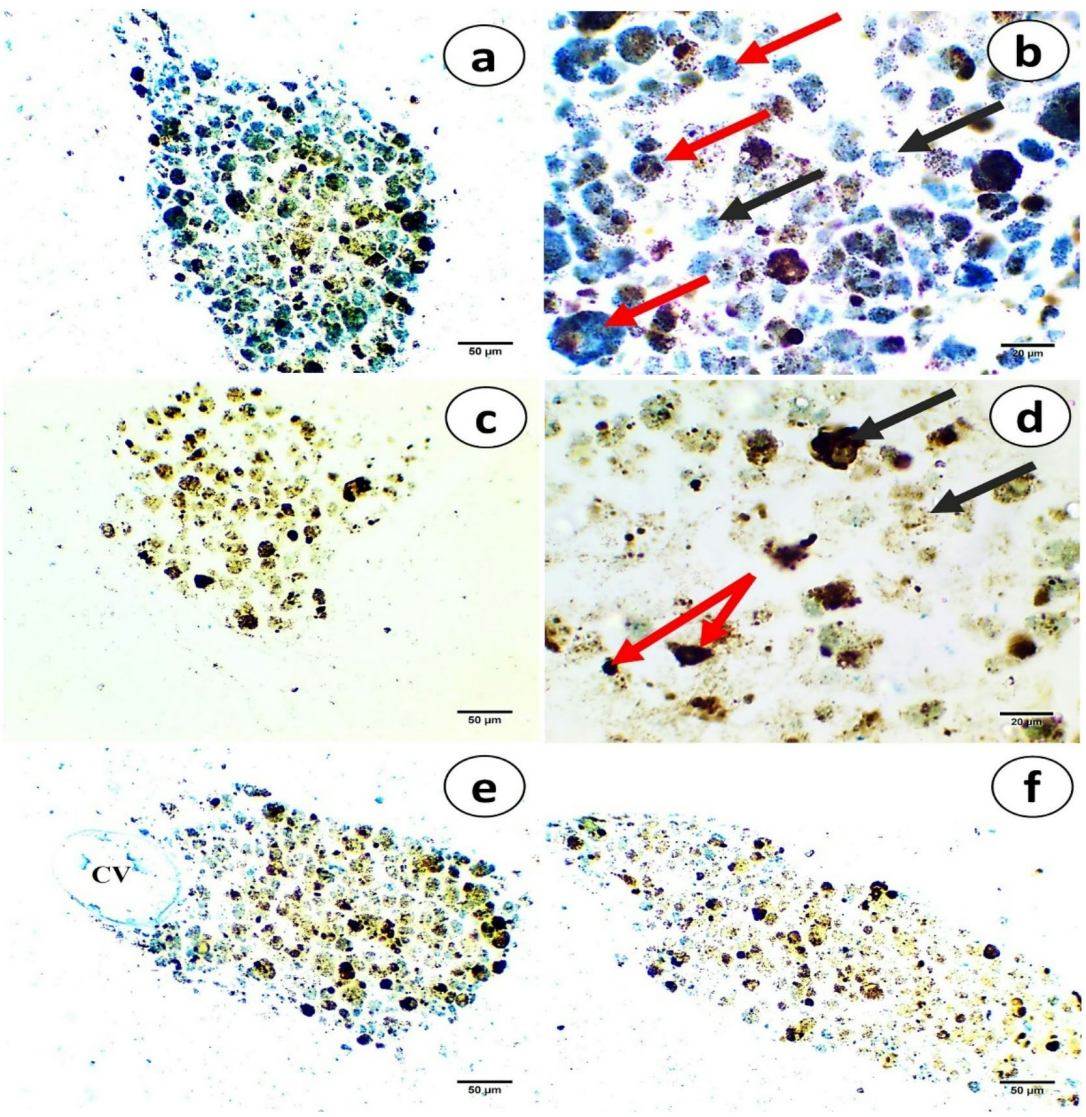
Photomicrographs of liver tissue sections of *M. kannume* stained by perls’ technique to visualize different pigmented granules in MMCs showing **(a)** increased hemosiderin deposition, **(b)** hemosiderin contains blue granules (black arrows), hemosiderin encloses blue and brown granules (red arrows), **(c)** increased lipofuscin deposition, and **(d)** lipofuscin contains brown granules and few ones enclosed black granules (black arrows). Few, dense, and hemogenous melanin pigments (red arrows), **(e)** increased lipofuscin deposition, and some hemosiderin are concentrated adjacent to the central vein and at MMC boundaries **(f)**. X40 **(a**,** c**,**e**,** f)** and X100 **(b**,** d)**.

Liver sections are stained with Perls’ technique to visualize different types of macrophage pigments within MMCs (Fig. 4). The first MMCs observed showed more hemosiderin, appearing as predominantly large, blue granules. These cells contain numerous heterogeneous cytoplasmic granules; in some, aggregations of fine blue deposits are observed, whereas others exhibit both fine blue deposits and a few coarse brown granules **(**Fig. 4a and b**)**.

Other MMCs predominantly contain lipofuscin, which is characterized by its brown coloration. This pigment appears as fine brown granules, with occasional homogeneous black granules interspersed within the cytoplasm. Melanin pigments are observed with comparatively smaller numbers, appearing as small, homogenous, and dense black granules **(**Fig. 4c and d**)**. Furthermore, hemosiderin pigments are concentrated adjacent to the central vein and at MMC boundaries **(**Fig. 4e and f**)**.

**Fig. 5:**
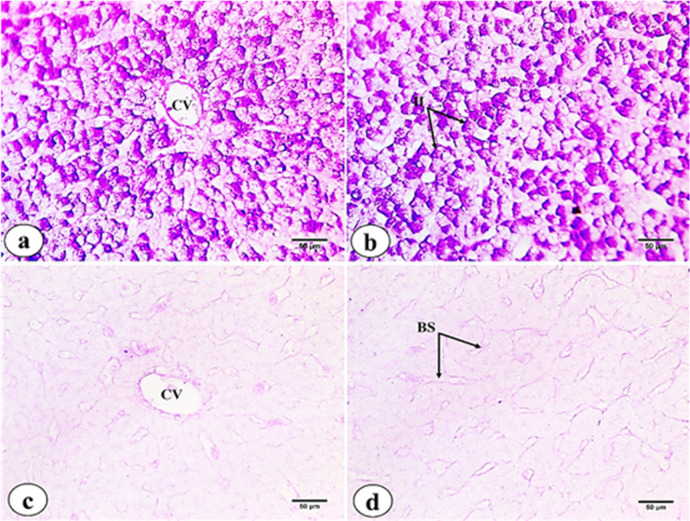
Photomicrographs of liver tissue sections of *M. kannume* stained with PAS showing the distinctive distributions of polysaccharides in hepatic tissue. **(a)** Structural polysaccharides in the endothelium of the central vein (CV). **(b)** Glycogen deposits (stored polysaccharides) in the periphery of hepatocytes (H), indicating glycogen flight. **(c)** Glycogen was digested in hepatocytes (H), and structural polysaccharides remained around the central vein (CV). **(d)** Structural polysaccharides remained in the basement membrane of blood sinusoids (BS) (X40).

PAS staining exhibited variations in the distribution of structural polysaccharides and reserve glycogen within hepatic tissues. Most hepatocytes show strong positive reactions with homogeneous glycogen deposits (glycogen flight). Conversely, the central vein exhibits a faint positive reaction for polysaccharides in its endothelial lining and the encompassing connective tissue sheath. Also, blood sinusoids exhibit a faint positive reaction for polysaccharides **(**Fig. 5a and b**)**. This is confirmed by treatment with human saliva, which completely digests glycogen stored in hepatocytes. Subsequently, only structural polysaccharides remained visible around the central vein and in the basement membranes of blood sinusoids **(**Fig. 5c and d**)**.

**Fig. 6:**
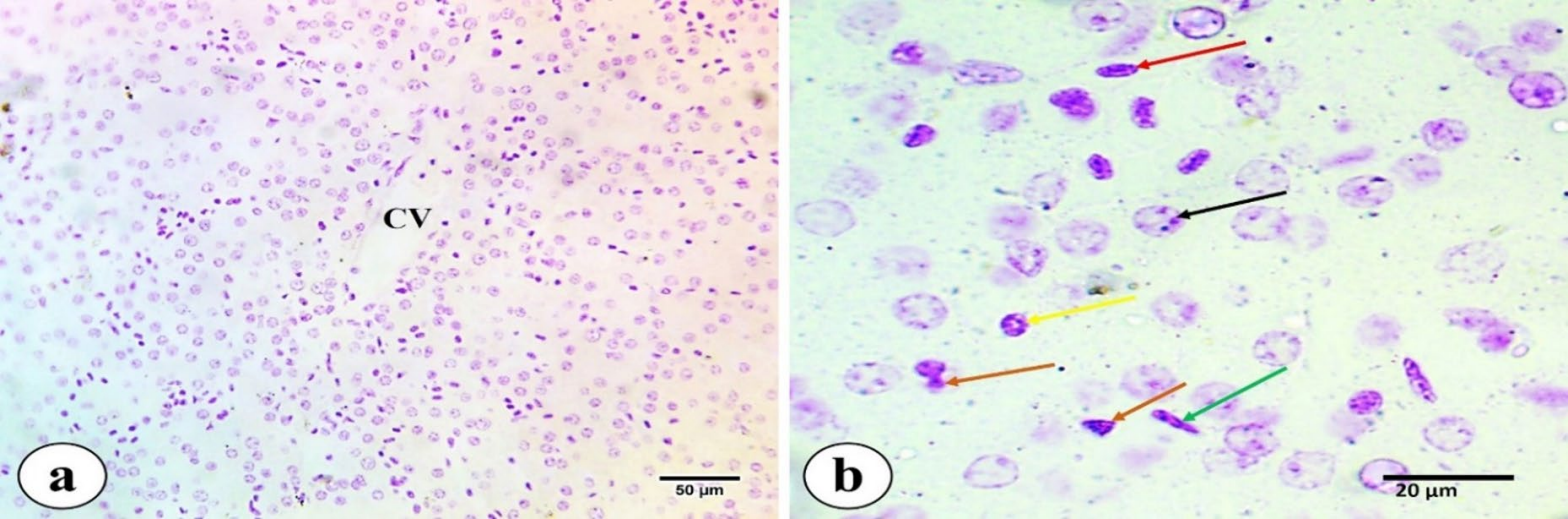
Photomicrographs of liver tissue sections of *M. kannume* stained by the Feulgen reaction showing: **(a-b)** different shapes of nuclei (N) of hepatic tissue. N. of red blood cells (red arrow), N. of hepatocytes (black arrow), N. of lymphocytes (yellow arrow), N. of Kupffer cells (orange arrow), N. of endothelium (green arrow). X40 **(a)** and X100 **(b)**.

Picrosirius red stain reveals the distribution of collagen fibers in hepatic tissues of *M. kannume*. A weak positive reaction is observed in the hepatic capsule and around the boundaries of blood sinusoids, indicating few collagen fibers **(**Fig. 6a**)**. In contrast, a high positive reaction is noted around the central vein, revealing more collagen fibers, while delicate collagen fibers surround the MMCs **(**Fig. 6b**)**. In liver sections stained with orcein, elastic fibers are less prominent type of connective tissue in the liver. Pale positivity reaction in the hepatic capsule, indicating very scarce distribution of elastic fibers **(**Fig. 6c**)** and more elastic fibers surrounding the central vein **(**Fig. 6d**)**.

**Fig. 7:**
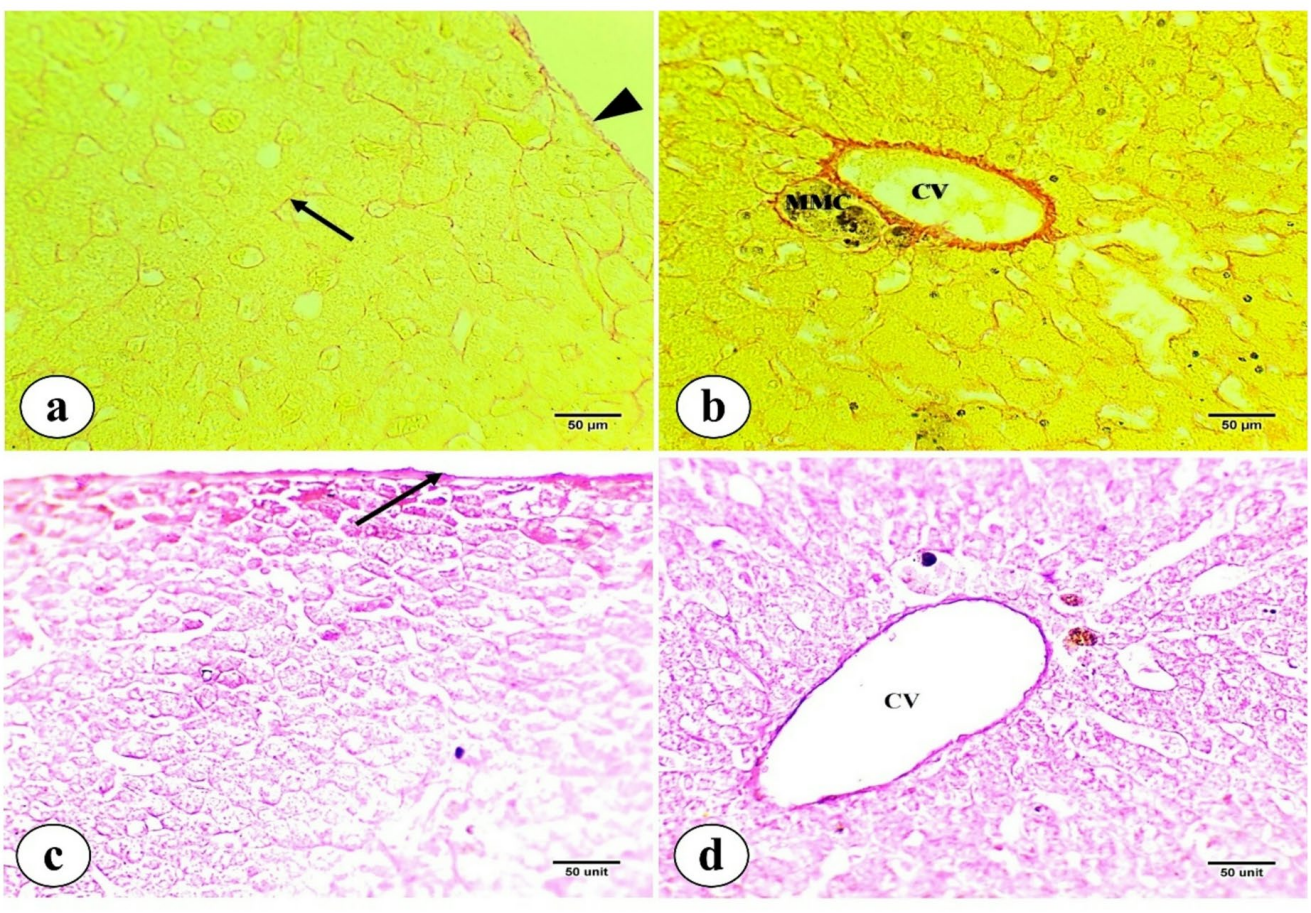
**(a**,** b)** Photomicrographs of liver tissue sections of *M. kannume* stained by picrosirius red stain showing the normal distribution of collagen fibers. **(a)** Few collagen fibers are distributed in the hepatic capsule (headarrow) and around blood sinusoids (arrow) and **(b)** more collagen fibers are observed around the central vein (CV). **(c**,** d)** Photomicrographs of liver tissue sections of *M. kannume* stained by Orcein stain for demonstration of elastic fibers showing: **(c)** scarce elastic fibers found in the hepatic capsule and **(d**) more elastic fibers distributed around the central vein (CV) (X40).

The Feulgen reaction reveals various nuclear morphologies in liver sections of M. kannume **(**Fig. 7a**)**. Hepatocyte nuclei appear large and vesicular, with a dispersed chromatin pattern. A strong positive reaction is observed in the periphery of the nuclear envelope (peripheral chromatin). Furthermore, different nuclear shapes are noted: endothelial cells of blood sinusoids exhibit elongated, spindle-shaped nuclei; Kupffer cells display irregular nuclei; red blood cells contain ovoid nuclei; and lymphocytes show small, rounded nuclei. The chromatin in all observed nuclei is dispersed, indicative of the chromatin network of interphase nuclei **(**Fig. 7b**)**.

## Discussion

*M. kannume* is considered one of the most interesting fish in the Nile with commercial importance. The present study investigated serum biochemical parameters and the histological structure of the liver, an important metabolic organ that serves as a biomarker of fish health, nutrition, and water balance^56,88^. This study aimed to provide baseline information for *M. kannume*. Moreover, normal blood parameters for individual fish species enable future systematic comparisons between species and assessment of their adaptation capacity to environmental conditions^15,36,38^.

The present results dealt with the basal levels of glucose, total protein, cholesterol, and liver enzymes (AST, ALT, and ALP) as important serum biochemical parameters in the fish. Previous similar studies demonstrated these biochemical parameters on different species of the same order (Osteoglossiformes) as reported by^93,94^. Other similar studies were conducted on different freshwater teleost species, as reported by^46,58,88^, and^92^. The present results showed variations in blood parameters, which may be due to general health, age, fish diet, and sample tissue preparation^71,93^.

Biochemical characteristics of most fish species were examined to determine normal blood levels and ranges^40,89^. Accordingly, the ranges of serum biochemical parameters differ from species to species^55,81^. The glucose level in blood serum is the most reliable measure of fish stress^71^. Otherwise, total protein, cholesterol, and triglyceride levels are crucial biomarkers for the nutritional state of fish^52,75^.

In the present results, total protein, glucose, and cholesterol are within the normal ranges for control Nile species reported by Kandeepan^58^, Heiba et al.,^46^, and Soliman et al.,^88^. The values of total protein are slightly lower than those described in many earlier studied fish, such as *Oncorhynchus mykiss*^76^, *Rhamdia quelen*^21^, *Arapaima gigas*^93^, *Mugil cephalus*^80^, *Diplodus noct*^83^, and *Oreochromis niloticus*^3^. These variations are due to differences in protein levels among teleost species^91^. Glucose levels have been attributed to stress resulting from low oxygen levels, manipulation, and diet, as well as to branchial damage. However, glucose levels fluctuate, glucose is also essential for brain function^87^, suggesting the existence of a glucose-sensing system in fish. Our results indicate relatively low glucose levels. Higher values are characteristic of active fish, whereas lower values are reported for less active bottom-feeders as *M. kannume*^47,62^. The lipid represents typically the economical form of energy storage in fish, deposited in various organs throughout the body^43^. The cholesterol level of *M. kannume* showed some variations compared to previous studies on other fish species. Thus, the cholesterol plasma levels vary according to age, general health conditions, and fish feeding diet^93^. ALT, AST and ALP enzymes are considered indicators of integrity and function of the liver^2,48^. According to Garai et al.,^41^ and Paulusma et al.,^70^, these enzymes play a vital role in the metabolism of amino acids and carbohydrates. In the current study, fluctuations were detected in AST, ALT and ALP levels. Such variations were detected in other fish (Nicula et al., 2010).

This study describes, for the first time, the normal anatomy and histology of the liver in *M. kannume*. The liver anatomy showed clear histological differences from that of other teleost species. The liver location of *M. kannume* resembles that of other teleosts^34,68,90^; however, hepatic lobes showed some differences in their shape and number. Therefore, it adapts to the shape and size of the internal cavity, as well as the space occupied by other visceral organs. Moreover, the weight, size, and volume of the liver vary depending on the animal’s body length and weight^4,18,44,78^. Furthermore, the liver of our studied fish appeared reddish brown in color, which may be due to rich vascularity, indicating that the fish was in normal health and in good nutritional condition, as described in almost all other carnivorous fish^4,74,97^.

In the current study, the liver parenchyma of *M. kannume* was not divided into lobules like other teleosts^97^;^34,67,72^, but a portal area was present and formed of a bile duct, which was lined by a simple layer of cuboidal cells and the portal vein. The biliary tract was classified into four types according to^9^: isolated type, biliary-venous tract (BVT) type, biliary-arteriolar tract (BAT) type, and portal-tract type. Our study showed that the biliary tract in M. kannume belongs to the biliary-venous tract (BVT) type, as in *Inimicus japonicus*^9^ and molly fish^53^. In contrast to many telestei, we observed that the biliary canaliculus originates between three and four hepatocytes. Variations in the structure of the biliary system are related to dietary patterns and are adapted to hepatic function. Through the bile system, toxicants are neutralized and removed from the liver^9^.

Hepatocytes were arranged in anastomosing cords radiating between the central vein and portal area, forming interconnected networks. This histological feature of the liver is similar to that described in *Hypostomus francisci*^78^, in molly fish^53^, in *Sparus aurata*^33^, in *Astyanax altiparanae*^24^, in *Pangasius sanitwongsei*^84^, and in *Diodon hystrix*, *Arothron hispidus*, and *Pseudobalistes fuscus*^13^.

The hepatocyte-sinusoidal structure is very important because hepatocytes absorb molecules from sinusoids and secrete essential macromolecules (e.g., lipoproteins, albumin, fibrinogen). In the cord-like structure, hepatocytes are closely associated with sinusoidal capillaries, forming a dense network, as in mammalian livers, which exhibit high metabolic activity^9,66^.

Hepatocytes were polyhedral cells with centrally located and rounded nuclei for most fish species, as reported by El-Bakary and El-Gammal^33^, Monsefi et al.,^65^, Sayrafi et al.,^84^, and Sales et al.,^78^. They are specialized in the storage of reserve components, mainly glycogen and lipids^34^. In contrast, their nuclei differ from those of tetraodontiform fish, which are peripherally located because the cytoplasm contains a high concentration of lipids^13^. Kupffer cells were distinguished in hepatic sinusoids of *M. kannume* in different shapes, unlike other fish livers. These cells have phagocytic activity to eliminate pathogens that enter the liver via the bloodstream, as reported by Shwartz et al.,^86^.

MMCs play an important role in the immune system through phagocytosis, destruction, detoxification, and the recycling of exogenous and endogenous materials^8,77^. The present investigation revealed the occurrence of MMC aggregations in the liver, located at various sites associated with hepatic structures. These results are in agreement with those observed in *Geophagus brasiliensis* and *Hoplias malabaricus*^78^, in tetraodontiformes^13^, in *Cynoscion guatucupa*^28^, and in *Hemisorubim platyrhynchos*^8,34^.

On the other hand, MMCs in *Hoplias malabaricus*^61^ and in *Leporinus macrocephalus*^20^ have been recorded only beside blood vessels. Otherwise, in *Hypostomus francisci*, melanomacrophages were dispersed^78,98^, and in the Clupeiformes and Salmoniformes, MMCs were less organized and harder to characterize^8,79^.

We recorded different pigments in MMCs, including yellow, brown, and black, as observed under H&E staining. However, in Perls’ technique, they exhibited blue, brown, and black colors. The study of these pigments reflects the role of macrophages in metabolic activities^60^. Melanin is important in neutralizing free radicals and other toxic substances produced by the degradation of phagocytized cellular debris. As well as its role in the synthesis of antibacterial complexes such as hydrogen peroxide^31^. Lipofuscin is a product of oxidation of polyunsaturated fatty acids and protein oxidation, and its levels increase in response to aging and tissue damage^50^. Hemosiderin originates from the degradation of damaged erythrocytes or from excessive iron accumulation within tissues. This pigment is associated with haemolytic anaemia and prolonged starvation^8,78^. In the current study, hemosiderin and lipofuscin were homogeneously distributed, whereas melanin formed clusters within MMCs; these results agreed with those of Dang et al.,^27^.

Previous studies^77] and [29^ described lipofuscin as the most abundant pigment in MMCs of teleost liver, which is consistent with our findings. However, certain examined sections exhibit variations with hemosiderin appearing more abundant than other pigments. A similar result was reported by Agius and Roberts^8^ and Manrique et al.,^64^, who explained deposition of hemosiderin in MMCs may be associated with the phagocytosis of excess iron.

Fish liver is the primary source of carbohydrates, which are essential nutrients for proper function and the major source of blood glucose. Blood glucose is stored in the liver in the form of glycogen^25^. *M. kannume* hepatocytes were PAS-positive, containing relatively large amounts of glycogen dispersed peripherally in the cells, which explained why glucose was not high in blood compared with other fish species, such as *Arapaima gigas*^93^, *Mugil cephalus*^38^, *Osteoglossum bicirrhosum*^94^, and *Oreochromis niloticus*^88^. These variations in the glycogen-to-glucose ratio among species may be related to swimming behavior, the nutritional status of fish, and biological and environmental factors^25^. Active fish showed large glycogen deposits, whereas sedentary fish had large lipid reserves (Akiyoshi et al., 2001).

Collagen fibers are the primary structural protein in the extracellular matrix (ECM) of connective tissue in the body^11^. In the current investigation, the liver is encapsulated by a loose connective tissue, consistent with observations in *Cetonopharingodon Idella*^10^, *Pangasius sanitwongsei*^84^, *Arothron hispidus*, *Abalistes stellatus*, *Diodon hystrix*, and *Pseudobalistes fuscus*^13^. This observation contrasts with findings in *Acipenser stellatus*^85^, in which the hepatic capsule is characterized by dense connective tissue. Picrosirius red staining confirmed the presence of abundant collagen fibers within the liver. Similar findings were also reported in^78^. Collagen increases in the liver when exposed to toxic substances that cause fibrosis and hepatic degeneration^14^. The liver stained positively with orcein, which reflects the presence of elastic fibers in the liver capsule and around blood vessels; these fibers provide elasticity^49^.

The Feulgen technique exhibited magenta color in the nuclei. Robert Feulgen and Heinrich Rossenbeck proposed Feulgen’s methods for detecting DNA as a component of chromatin and chromosomes about a century ago^19^. We could investigate various distributions of chromatin in hepatic tissue cells. Previous studies, such as Rao^74^, used this method in the investigation of DNA in normal and infected liver cells of *Channa punctata*.

## Conclusion

In conclusion, the present research provides baseline physiological parameters for *M. kannume* and liver enzymes. It conducts a comprehensive study of liver histology using various histological and histochemical techniques, emphasizing its architecture, which is optimized for distinct functions. Liver of *M. kannume* is composed of hepatocytes with a cord-like structure, sinusoids, and bile cuniculi, which fascinate with its vital role in metabolism, detoxification, and production of bile. Furthermore, the typical appearance of MMCs in different shapes and locations is considered an important biomarker of water quality and a stressful environment.

## Data Availability

All data generated or analysed during this study are included in this published article.
